# Nanoscale effects in the characterization of viscoelastic materials with atomic force microscopy: coupling of a quasi-three-dimensional standard linear solid model with in-plane surface interactions

**DOI:** 10.3762/bjnano.7.49

**Published:** 2016-04-15

**Authors:** Santiago D Solares

**Affiliations:** 1 Department of Mechanical and Aerospace Engineering, George Washington University, Washington, DC 20052, United States

**Keywords:** atomic force microscopy, modeling, polymers, simulation, spectroscopy, standard linear solid, surface elasticity, surface energy, viscoelasticity

## Abstract

Significant progress has been accomplished in the development of experimental contact-mode and dynamic-mode atomic force microscopy (AFM) methods designed to measure surface material properties. However, current methods are based on one-dimensional (1D) descriptions of the tip–sample interaction forces, thus neglecting the intricacies involved in the material behavior of complex samples (such as soft viscoelastic materials) as well as the differences in material response between the surface and the bulk. In order to begin to address this gap, a computational study is presented where the sample is simulated using an enhanced version of a recently introduced model that treats the surface as a collection of standard-linear-solid viscoelastic elements. The enhanced model introduces in-plane surface elastic forces that can be approximately related to a two-dimensional (2D) Young’s modulus. Relevant cases are discussed for single- and multifrequency intermittent-contact AFM imaging, with focus on the calculated surface indentation profiles and tip–sample interaction force curves, as well as their implications with regards to experimental interpretation. A variety of phenomena are examined in detail, which highlight the need for further development of more physically accurate sample models that are specifically designed for AFM simulation. A multifrequency AFM simulation tool based on the above sample model is provided as supporting information.

## Introduction

The accurate characterization of viscoelastic materials with atomic force microscopy (AFM) is of high interest [1–16], but it is also a very difficult task due to the complexity of the material behavior phenomena that govern the AFM observables. In contrast, the description of the bulk behavior of viscoelastic materials can frequently be represented with relatively simple models combining linear springs and dampers [14], which are appropriate for the specimen geometries used at the continuum scale. A common example is a viscoelastic film trapped between two parallel plates that are displaced with respect to one another while the stress in the material is measured [17–18]. Drawing a connection from continuum mechanics to AFM measurements (where the probe geometry is relatively simple, often assumed spherical) may appear to be straightforward at first glance, but a detailed analysis reveals specific complexities that can make quantitative accuracy an elusive goal. For example, in contrast to a typical bulk experiment, the stresses and strains imposed by the AFM probe on the sample are not uniformly distributed throughout the material, but are often localized near the axis of the indentation. Second, at the scale of an AFM measurement, the surface layer mechanical properties (which often differ from the bulk properties, as discussed below) may play a prominent role. Specifically, as the tip compresses the sample, it is easy to imagine how the indentation leads to an increase in the sample surface area, which has an associated energy cost either due to elasticity or surface free energy, or both [19–20]. The overall effect is that as the tip travels into the surface, the surface profile evolves in a way that minimizes the associated energy cost. Third, these surface free energy and elastic energy effects are often neither isotropic nor uniform, as there is generally a variation in the structure and morphology of most viscoelastic surfaces (e.g., polymers) in the horizontal direction (examples are provided below). Furthermore, at the scale of an AFM indentation it may be difficult to properly define the surface energy (even conceptually) since the local material constituents (e.g., polymer chains) can significantly differ from their neighbors and can be, to a varying degree, flexible and mobile. At this scale, the surface is not a smooth continuum but may instead contain molecules oriented in various directions, as well molecules trapped in conformations that do not correspond to a global energy minimum (especially in the compressed volume directly under the AFM tip). Additionally, the behavior of the molecules on the surface may be influenced by chemical or dispersion (van der Waals) forces exerted by the probe, thus leading to a situation in which the sample is influenced by the measurement itself. One could continue extending the list of phenomena that preclude an ideal measurement by considering other issues such as limitations of the measurement method and instrumentation, almost arriving at the conclusion that it is nearly impossible to carry out quantitatively accurate measurements of viscoelasticity with AFM, unless one assumes that the sample follows the simplest continuum behaviors.

For the most part, the development of new AFM imaging and spectroscopy methods has relied on continuum assumptions that enable the construction of well-defined surface models. This is quite reasonable, and in the absence of elaborate surface models that can easily be incorporated into the methods, this has been a very fruitful course of action. For example, within contact-resonance AFM (CR-AFM) methods (including dual-amplitude resonance tracking, DART) [2–6], the surface is treated using a linear Kelvin–Voigt model, which consists of a linear spring in parallel with a linear damper. Linear models are used in this case because the oscillation amplitude of the AFM tip is very small, so the tip–sample interaction force curve at the desired force setpoint can be considered to be a straight line for the range of tip positions explored. Similar approaches have been used in force modulation techniques (FMOD-AFM), where the sample is dynamically probed at frequencies well below the cantilever resonance frequency [21]. Novel spectroscopy methods have also been recently developed for intermittent-contact imaging. For example, it is now possible to extract tip–sample force curves using dual-eigenmode frequency-modulation AFM [10] and intermodulation AFM [11–12]. In these methods, the surface is modeled as a continuum material with a well-defined Young’s modulus, which interacts with a spherical AFM probe and is assumed to dissipate energy in proportion to the probe’s instantaneous velocity and depth of indentation. Here, an analytical description of the force as a function of tip position and velocity, 
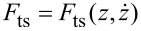
, is postulated, and the experiment is performed with the objective of extracting the model constants. Despite the simplicity of the surface models available, these methods clearly represent very important milestones in the development of AFM spectroscopy and already have a wide range of practical applications. Nevertheless, these rapid AFM developments also call for additional efforts at the other end of the research problem, namely the development and implementation of more physically accurate surface models that are able to describe nanoscale surface complexities such as those discussed in the previous paragraph.

With the above in mind, the objective of this paper is to carry out a qualitative exploratory study of the coupling of viscoelastic behaviors with nanoscale surface effects within a tip–sample model that can be used for the simulation and interpretation of AFM experiments. The work departs from a previously introduced quasi-three-dimensional (Q3D) implementation of the standard linear solid (SLS) model for representing viscoelastic surfaces [13–14,22–24] and considers the enhancement of the model through the incorporation of in-plane elastic effects within the top surface layer. The study highlights measurement interpretation challenges and physical phenomena that have not yet been addressed in depth within AFM. The article is organized as follows: first a thorough presentation of the SLS model is provided, describing its elements, constitutive equations, corresponding complex modulus, and the relationship between its parameters and those one would use in simulating AFM imaging. Next, a detailed discussion of surface effects is offered, followed by a description of the enhanced surface model. A Results and Discussion section follows, which treats specific numerical examples focusing on the tip–sample force curve features, indentation profiles, tip geometry effects and their interaction with one another. The next section consists of a description of the software tool, whose source code and input file are provided as supporting information. The paper closes with the section Conclusion.

The author cautions the reader that although the calculations and discussions presented here can be useful in highlighting future research directions regarding the development of AFM methods and models for viscoelastic materials, all the analyses are based on linear material behaviors that may fall short in the treatment of specific problems. Consider, for example, a tapping-mode experiment, which may involve local stresses and strains that are too large to be treated linearly, or local heating and melting of the sample, which can cause the material properties to vary with time and location within the sample. This paper, therefore, offers only a glimpse into the research gaps that exist in the treatment of sample material properties within AFM simulation.

### Viscoelasticity and the standard linear solid

**Figure 1:**
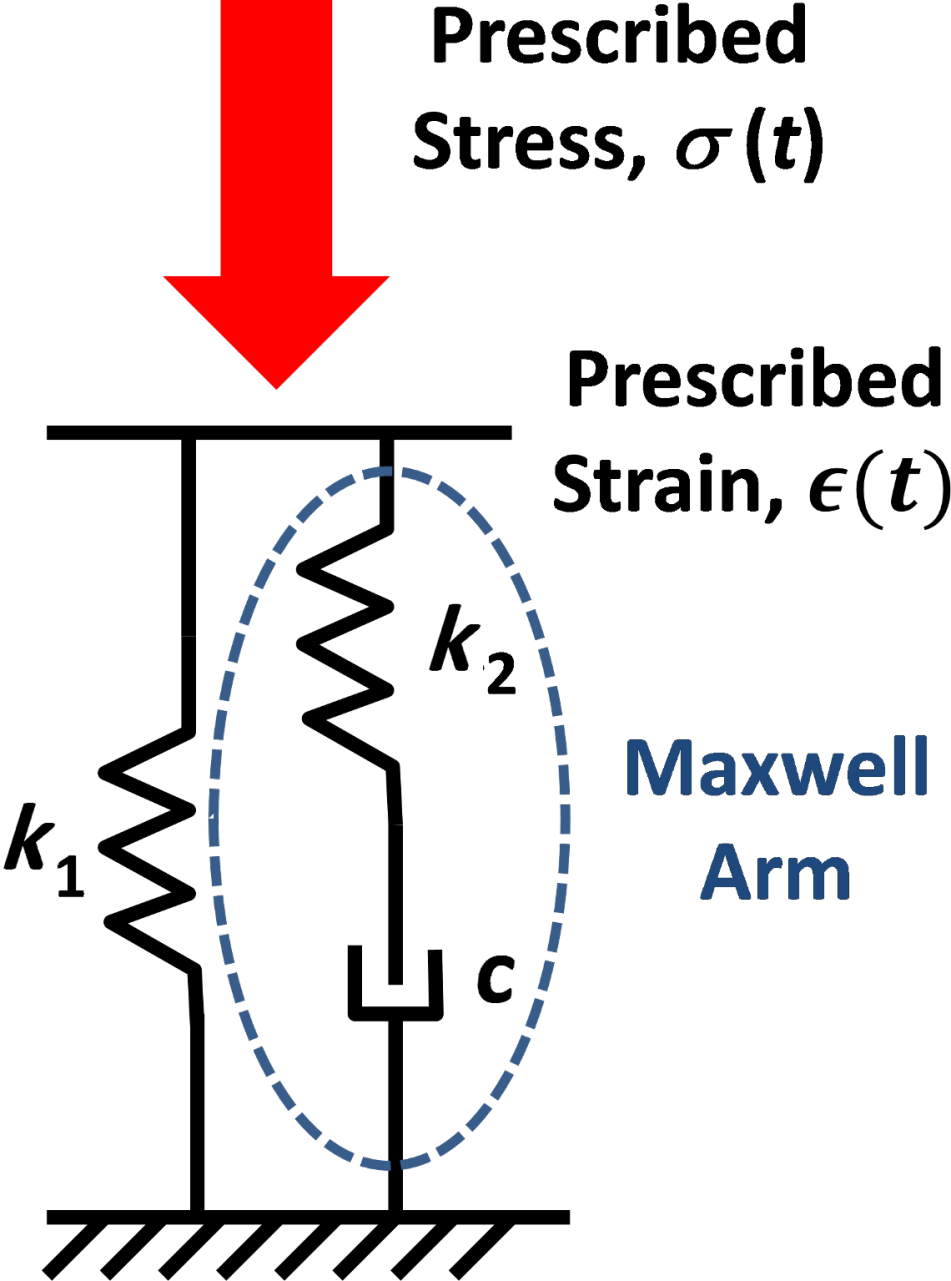
Standard linear solid model. The response of the model generally consists of a time-dependent stress resulting from the application of a prescribed strain trajectory or a time-dependent strain resulting from the application of a prescribed stress trajectory.

The standard linear solid (SLS) model is shown in Figure 1. It consists of a linear spring in parallel with a Maxwell arm, which in turn consists of a linear spring in series with a linear damper [13–14,25]. The SLS is the simplest viscoelastic model that is capable of reproducing the most fundamental viscoelastic behaviors, namely creep and stress relaxation [13–14,18,25]. There exist simpler models [13], such as the Maxwell model by itself (described above) and the Kelvin–Voigt model, which consists of a linear spring in parallel with a linear damper (this model is used in CR-AFM [3]). However, in the former model, the surface is unable to restore itself to its original state (it remains permanently deformed upon the application of a stress), and the latter model does not exhibit stress relaxation.

The SLS model was previously used in AFM simulations and its general properties and response have been extensively discussed [13–14]. Here the derivation of its constitutive equation [25] and the expressions describing its complex modulus are discussed [17–18], which offer a theoretical connection to experimental bulk measurements.

#### SLS constitutive equation

To derive the SLS constitutive equation [25] relating stress and strain, focus is placed on the Maxwell arm, which contains spring *k*_2_ and damper *c* (see Figure 1). It should be noted that the stress in this Maxwell arm, σ_m_*,* is the same for both of its elements because they are in series:

[1]
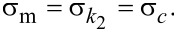


In contrast, the strain is additive:

[2]
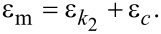


The above equation can be differentiated to give

[3]
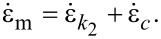


Since the spring *k*_2_ is linear, its stress–strain relationship is given by

[4]
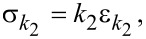


which can also be differentiated with respect to time to give

[5]
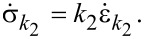


By definition of the linear damper, we also have

[6]
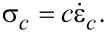


The substitution of Equation 5 and Equation 6 into Equation 3 gives

[7]
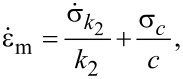


which, using Equation 1, can be rewritten as

[8]
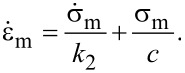


Since the Maxwell arm in the SLS model is in parallel with spring *k*_1_, the strain of the Maxwell arm and of the spring *k*_1_ is the same, but the stresses are additive:

[9]
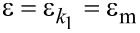


[10]
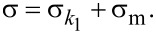


Differentiating Equation 10 gives

[11]
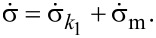


Solving for 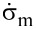 in Equation 8 and substituting it into Equation 11, along with the time derivative of the stress–strain relation for spring *k*_1_ (
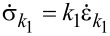
), and making use of Equation 9 to remove the subindices on the strains gives

[12]
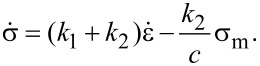


Finally, solving for σ_m_ in Equation 10 and introducing it into Equation 12 along with the stress–strain relation for spring *k*_1_ (
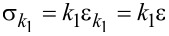
), the constitutive equation for the SLS model is found as:

[13]
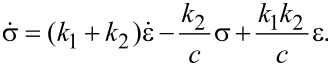


The above equation governs the response of the SLS model and will be used in the next section to derive expressions for its complex modulus. This value is measurable in an experiment that involves the application of a strain that varies sinusoidally in time, as long as the stress and strain are uniform throughout the specimen.

#### SLS complex modulus

The SLS parameters of a particular specimen can be obtained via the expressions for the model’s complex modulus (obtained from its constitutive equation), by fitting those expressions to the complex modulus behavior observed experimentally for the material under study. To derive the complex modulus of the SLS model, a periodic strain that varies sinusoidally in time with frequency ω is defined as [17–18,25]:

[14]
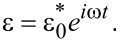


When the above strain is applied to the sample, it generates a sinusoidally varying stress of the form

[15]
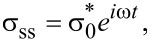


where both 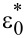 and 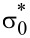 are complex (strictly speaking, 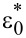 can be real in an experiment, since the phase of the applied strain can be defined as zero) and the subindex ss refers to the steady state condition after all transient responses have disappeared. The complex modulus is now defined as [17–18,25]

[16]
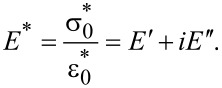


To find *E*′ and *E*″ Equation 14 and Equation 15 are substituted into the SLS constitutive equation, Equation 13. Then 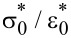 can be solved for, which can be written in the form of Equation 16 and gives

[17]
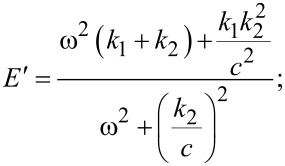


[18]
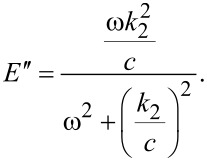


**Figure 2:**
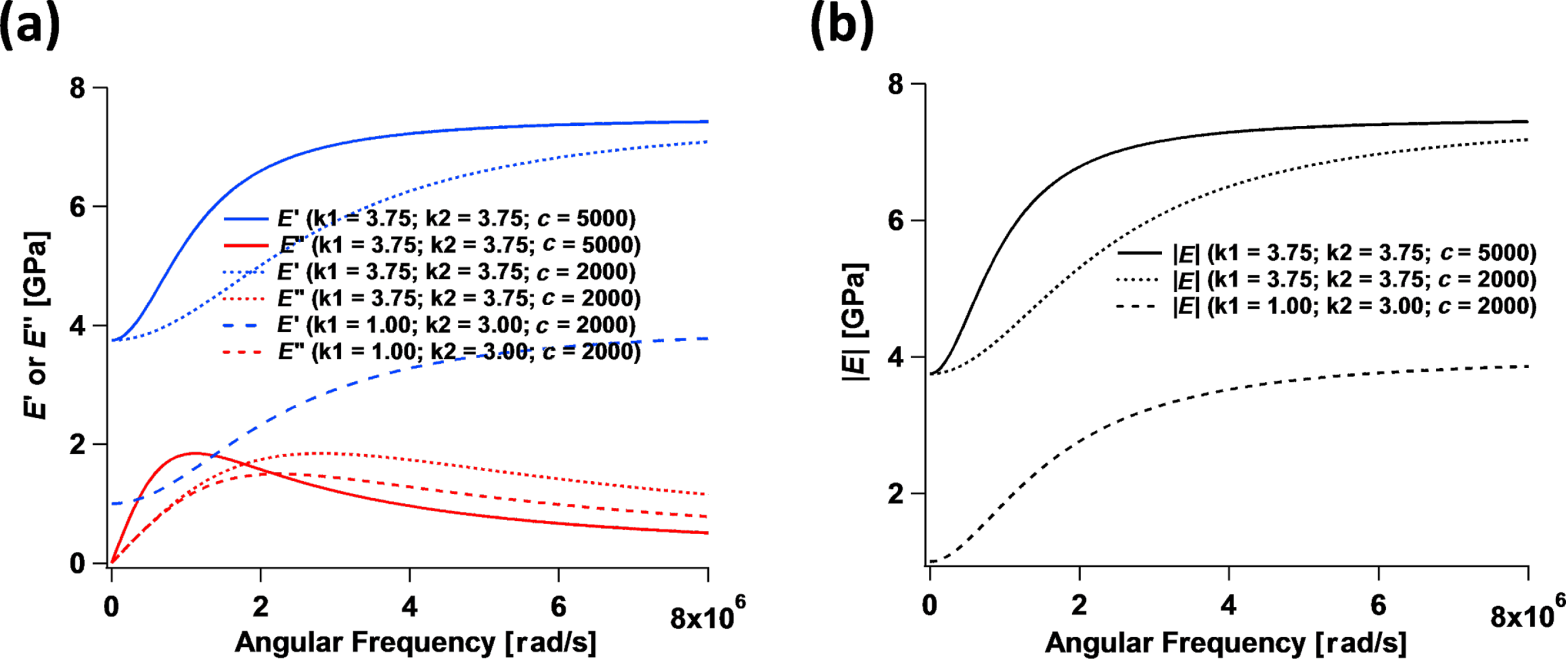
(a) Plots of the complex modulus components, *E*′ and *E*″ (Equation 17 and Equation 18, respectively), for the sets of model parameters indicated in the graphs. (b) Plots of the absolute value of the complex modulus corresponding to the traces shown in (a). The angular frequency axis is in radians per second, and the maximum axis value of 8 × 10^6^ rad/s corresponds to a frequency of approximately 1.27 MHz, which is nowadays not uncommon in AFM, especially within multifrequency methods [9].

*E*′ is associated with elastic interactions, while *E*″ is associated with dissipative interactions [25]. Figure 2 gives examples of plots of the above expressions as a function of frequency for a few sets of parameters. Of the measurable continuum quantities available, the complex modulus is the most appropriate to fit theoretical models to, and is also the most appropriate to draw a connection to within dynamic experimental measurement techniques such as AFM. In contrast, the Young’s modulus is not an appropriate measure because it is not well defined in a dynamic measurement (especially as the strain oscillation frequency is increased), and because in the case of viscoelastic materials, the stress and strain are not related by a simple constant as in the elastic case. Furthermore, it must be noted that the complex modulus is only applicable in the context of a continuous periodic measurement, such as the application of a sinusoidal strain (
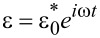
), as discussed above. Within the context of AFM, this condition can be approximately satisfied only in methods such as CR-AFM or FMOD-AFM [2–6,21]. Therefore, neither the use of the complex modulus nor of quantities derived from it (e.g., the loss tangent, which is the ratio of the loss modulus to the storage modulus) are appropriate for analysis of intermittent-contact AFM measurements (this includes both single- and multifrequency techniques) because, (i) the tip–sample interaction in that mode of imaging is not continuous and therefore the stress response of the material is not able to reach a steady state during the measurement, and (ii) the complex modulus depends on the frequency of the periodic sinusoidal sample deformation, which is not well defined in a tapping-mode experiment (see [26] for a discussion of discrepancies between intermittent-contact and contact-resonance viscoelasticity measurements). Furthermore, the spectrum of the force (stress) and displacement (strain) in intermittent-contact AFM contains a mixture of unknown and difficult to measure frequencies [15,27], and the experiment does not provide a spatial description of the strain distribution throughout the volume of the sample that is instantaneously interacting with the tip (not all sample differential volume elements that interact with the tip at a given instant undergo the same strain history, neither in terms of magnitude nor in terms of strain direction). This is why viscoelastic measurements with AFM are most commonly performed using CR-AFM or FMOD-AFM, where the sample is probed in contact-mode using a single oscillation frequency in the small-amplitude regime. However, even in these most ideal situations, there still exist challenges associated with the shape of the tip, which does not necessarily impart uniform compressive strain, but may cause both compressive and shear strains with an unknown distribution (within CR-AFM and FMOD-AFM measurements it is customary to assume that strains and stresses exist only in the normal direction, but this is only an approximation). Models of the type discussed here could aid in performing such characterizations with greater accuracy.

Regarding the quantification of elastic sample behaviors within periodic dynamic measurements, it is important to point out the two frequency limits for which the response of the 1D SLS model is purely elastic (see Figure 2). At zero frequency (or infinite deformation time scale) the complex modulus is equal to the storage modulus (the loss modulus is zero, so the complex modulus is real), which according to Equation 17 is equal to *k*_1_ and is called the rubbery modulus [25]. At infinite frequency the loss modulus also becomes zero and the complex modulus is again real and reduces to the storage modulus, but with a value of *k*_1_ + *k*_2_, called the glassy modulus [25]. Both of these moduli can be appropriate elastic constants to compare experimental AFM results to in the cases of very low or very high frequencies, if it can be approximately guaranteed that the stress and strain are uniform and their distribution is 1D.

#### Relationship between sample properties and AFM simulation parameters

Two very important considerations when relating AFM measurements or simulations to sample properties that are consistent with viscoelastic theory concern the uniformity of the deformation (as already stated above) and the units of the model parameters. In the above discussion of the 1D SLS model, it is assumed that the stresses and deformations are uniform throughout the specimen under study, and that the equations are written in terms of strains and stresses. This is why it is appropriate to speak of moduli (note that *k*_1_ and *k*_2_ are referred to above as moduli, and see also Equation 17 and Equation 18, as well as the discussion pertaining to them). However, in AFM one is generally concerned with displacements (indentation depths) and forces [16]. Therefore, in previous publications [14,28] *k*_1_ and *k*_2_ are defined as force constants with units of N/m and not as moduli with units of N/m^2^. However, to be consistent with the equations listed above, the relationship between the moduli and the linear force constants of the model are provided here.

**Figure 3:**
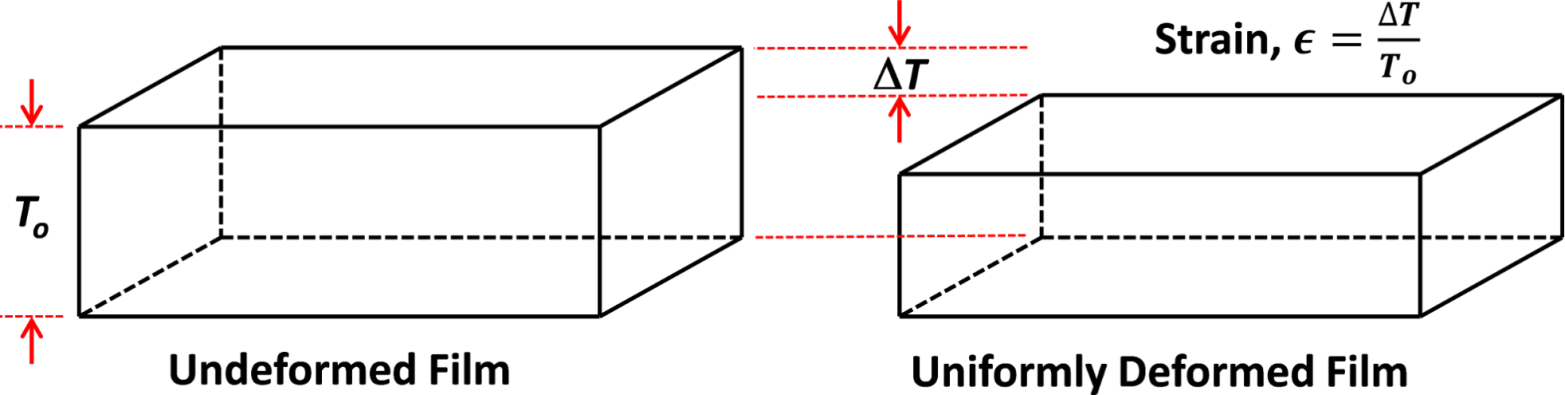
Illustration of the uniform deformation of a viscoelastic film of initial thickness *T*_0_. This type of uniform deformation is not attainable in AFM measurements conducted with standard tips.

Consider the viscoelastic film schematically drawn in Figure 3 and assume that its stress–strain relationship in the vertical (tensile–compressive) direction can be approximated using that of the SLS model (Equation 13). The undeformed film has an initial thickness *T*_0_ but it is then compressed uniformly across its entire area by a distance Δ*T*. If the film can be treated as a continuum, its strain can be defined as

[19]
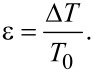


Since the film is described by the SLS model, the stress–stress relation of spring *k*_1_ (
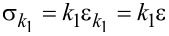
) can be used to write

[20]
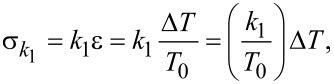


where 
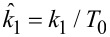
 (last term on the right hand side) is defined as a force constant normalized by surface area (with units of force/length^3^), whereby 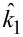 converts the displacement Δ*T* into the stress 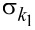. In previous AFM simulations [14,28] a force constant that is not normalized by unit area was simply given, such that multiplication by the displacement gives a force 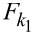 instead of a stress 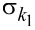. This is equivalent to assuming that the surface area, *A*, of the film being compressed under the AFM tip is known, since the total force is equal to the stress multiplied by the total area (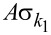 or 
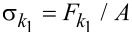
). In the code provided as supplementary information for this paper, the model input parameters are defined as 
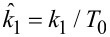
, 
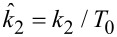
, and 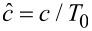 and have units of N/m/nm^2^, N/m/nm^2^ and Ns/m/nm^2^, respectively. The assumption of homogeneous stress and strain is violated in AFM measurements, where the deformation is minute compared to the dimensions of the sample and the deformation time scale can be short compared to the timescales required for full relaxation. In reality, the appropriate value of *T*_0_ to use in the calculations is not known in an experiment since the deformation may be limited to an unknown region near the surface (the deformed region can be localized, for example, due to cross-linking or layering of the material, which prevents the small deformation imparted at the top of the film from ever reaching the lower end of the specimen in a significant way). It is therefore not possible to establish a direct, rigorous connection between bulk SLS parameters and the SLS parameters to be used in an AFM simulation. Nevertheless, it is illustrative to carry out the exercise of converting model parameters to bulk properties under assumed ideal conditions. For example, consider a 50 nm thick film with 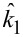 = *k*_1_ / *T*_0_ = 0.075 N/m·nm^2^ as used recently in [22]. In this case, *k*_1_ = 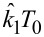 = 0.075 N/m·nm^2^ × 50 nm = 3.75 GPa, which is in the expected range for a typical polymer film investigated with AFM [10]. For the dissipation coefficient, *c* = 1 × 10^−7^ N s/m·nm^2^ × 50 nm = 5000 N s/m^2^ as was also used in [22].

### Surface effects in nanoscale probe measurements

Surface effects can emerge in AFM measurements when the surface area increases upon indentation by the tip, as this can generate forces in the plane of the surface which seek to reduce the surface area back to the original value. The restoring forces can be caused by the exposure of bulk molecules to the surface, where these molecules will no longer be surrounded by neighbors in all directions and will thus have a reduced number of favorable nearest-neighbor energy interactions [20]. The forces can also be due to elastic effects, such as in the case of a cross-linked or covalently bonded material, which will experience an internal restoring force without undergoing obvious changes in the arrangement of the molecules [19]. Depending on the material, the contribution of each mechanism to the total driving force counteracting increases in surface area can vary [19].

Some of the most striking examples of surface free energy effects are observed in covalent crystals, for which the creation of new surface area by splitting the crystal requires breaking covalent bonds and leaving dangling bonds on the surface [29]. Loosely speaking (that is, without considering reconstruction and/or relaxation), the surface energy correlates with the number of bonds broken per unit area, divided between the two surfaces that are created (the breaking of the bond requires that the appropriate bond energy be supplied and half of the energy cost is assigned to each of the two surfaces created). Often, surfaces undergo structural reconstruction upon the generation of new surface area in order to reduce the overall energy cost [29–31]. Even in the cases where there is no reconstruction, the surface still undergoes some type of relaxation, such that its final structure and specific energy differ from those of the bulk. The degree of crystallinity in viscoelastic surfaces can in general be low compared to that observed in crystals, and adjacent molecules are often not joined by covalent bonds [19]. Frequently, but not always, relatively large molecules are involved and their cohesive energy is governed by dispersion (van der Waals) and electrostatic forces, which are generally weaker than those generated by stretched covalent bonds or electrostatic interactions in ionic crystals, and which allow the molecules to rearrange with relative ease [19–20]. There are also many viscoelastic surfaces which are formed by macromolecules that have cross-linking covalent bonds, such as vulcanized rubber [19], in which case there are restrictions on the relative mobility of adjacent molecules.

**Figure 4:**
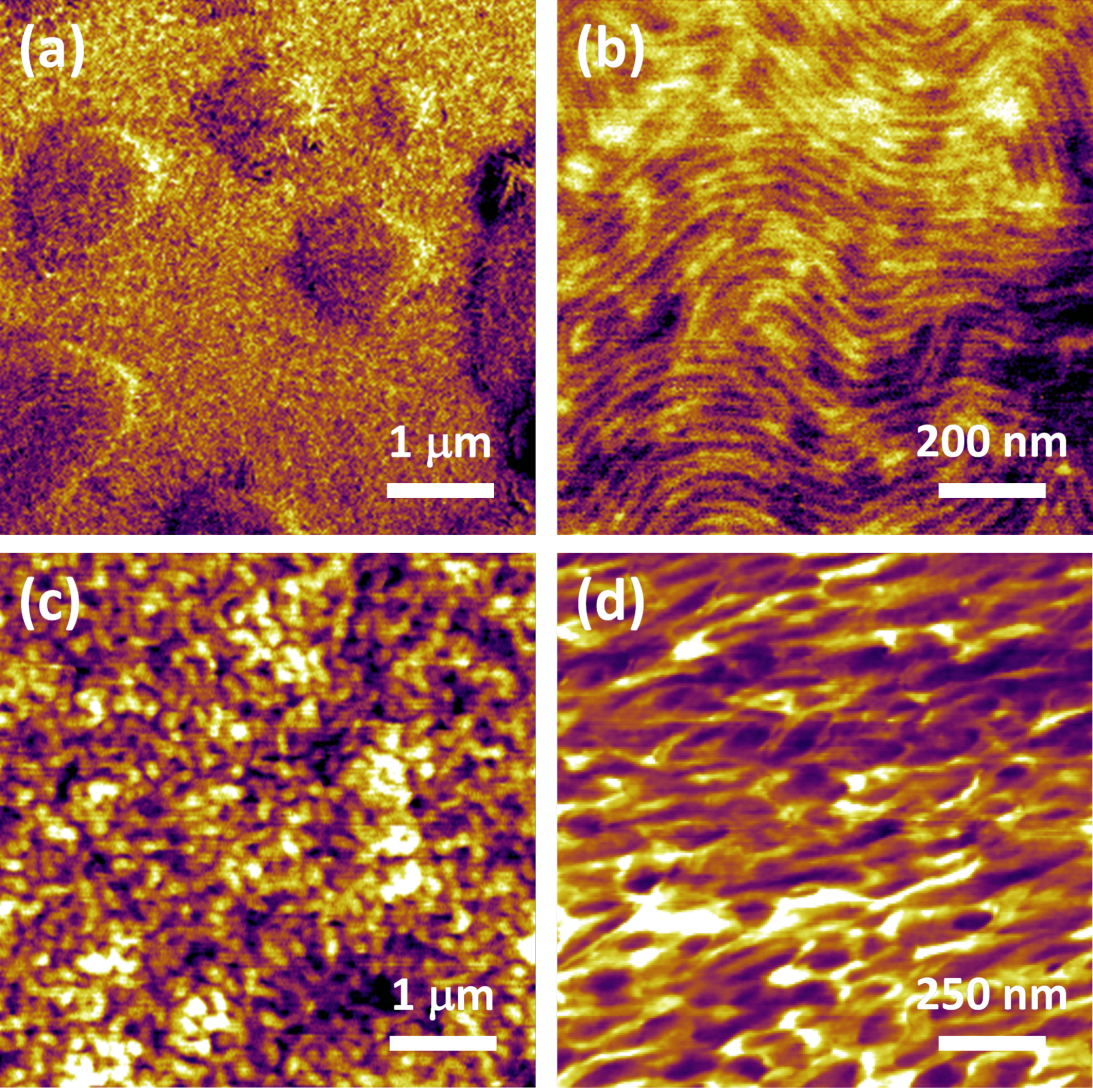
Examples of nanoscale polymer surfaces imaged with AFM. (a,b): Kraton (the height variation in (a) is 30 nm over a scan size of 5 × 5 μm); (c) Nafion (spin-coated thin film); (d) PEDOT:PSS. All images are phase images acquired in tapping-mode AFM. These images are only provided to illustrate typical polymer morphologies and are not related to the model implementation examples provided below.

From the above discussion, it is clear that the structure of the surface itself is not expected to be the same as that of the bulk, and that the surface structures of viscoelastic surfaces can be very complex and difficult to predict. Additionally, the constituent molecules in a viscoelastic material can exhibit some variation in size (molecular weight and structure), connectivity, distribution of functional groups, etc., such that they are not all necessarily identical [19]. Additionally, the types of surface configurations that are feasible depend on the structure of the source material (e.g., the monomers used and their proportion in a copolymer), the manufacturing process used, and the size (especially thickness in the normal direction) of the specimen under study [19]. For certain ranges of manufacturing conditions, precursor properties (e.g., the time–temperature history or molecular size), or dimensions, the material structure may be “trapped” into different configurations that do not necessarily correspond to a global minimum of energy [19]. Such complexity clearly offers a wider range of morphologies in comparison to a crystal. Figure 4 shows a few examples of AFM images of highly regular polymer surfaces that exhibit significant variability in the horizontal direction, thus precluding a rigorous continuum treatment at the nanoscale.

**Figure 5:**
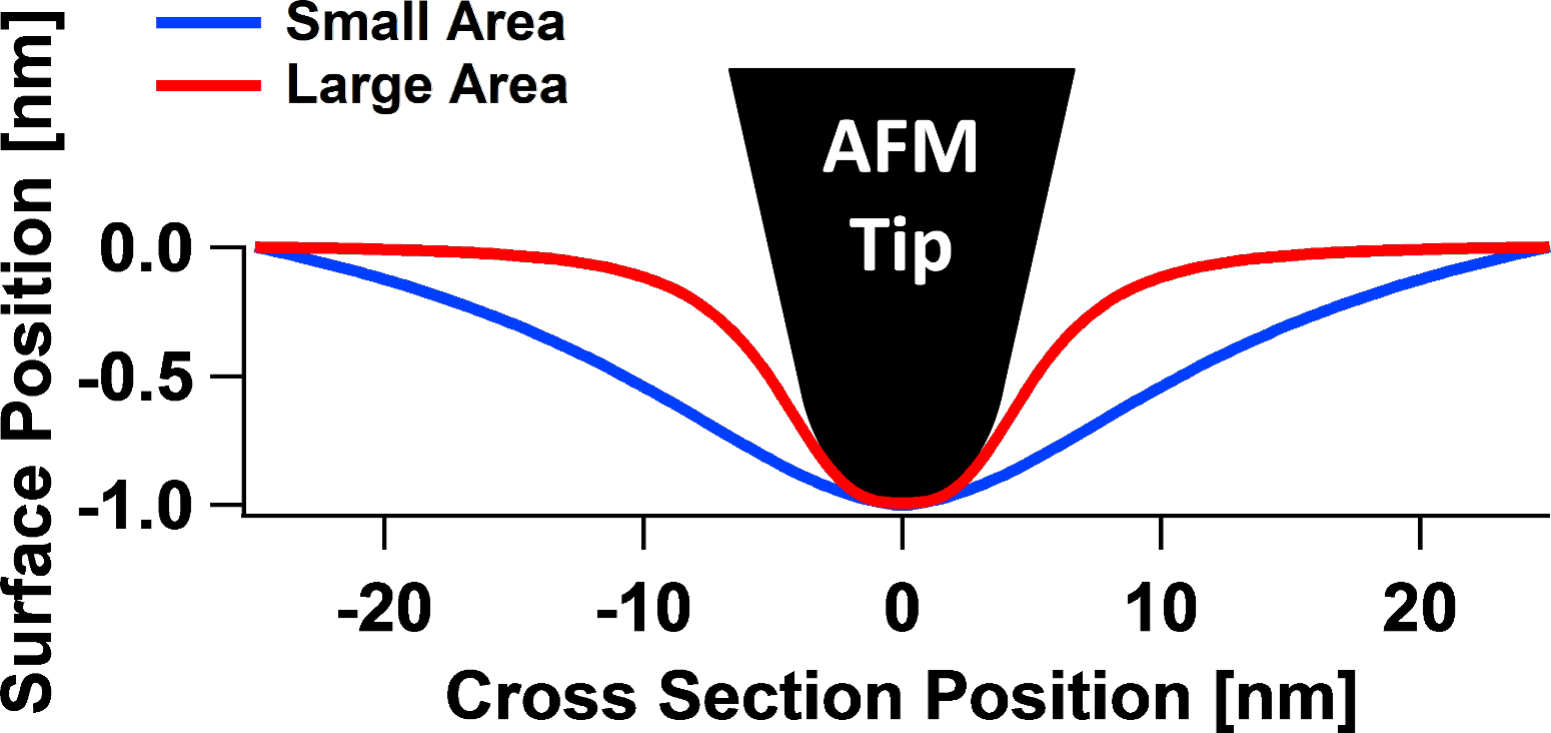
Schematic illustration of two surface profiles caused by indentation with an AFM tip. The blue profile corresponds to a smaller increase in surface area than the red profile.

The existence of a surface energy “penalty” either due to surface free energy or due to elasticity leads to an attempt on the part of the material to reduce its surface area. In the dynamic loading of macroscopic specimens this does not play a role because the surfaces generally remain unchanged (flat) [17–18], but this can be very important in an AFM experiment, where indentation by the tip leads to a curved surface (a curved depression embedded into an otherwise flat surface), whereby the total final area of the cavity created is a function of the curvature of the surface. This is illustrated schematically in Figure 5, which shows two examples of surface profiles caused by interaction of the surface with the tip. The blue profile shown corresponds to a smaller surface area than the red profile. Since the surface free energy and elasticity depend on the material and its configuration, different samples of different materials will exhibit different surface curvature profiles upon indentation by the same AFM tip. As a result, there will be different surface contributions to the total force acting on the AFM tip in each case. This phenomenon can be further complicated by the fact that the proximity of the AFM tip generally leads to favorable nonbonded interactions with the surface, which also affects the surface curvature. Furthermore, additional effects are expected for surfaces that are more cross-linked than the bulk material or surfaces that react with the environment, and also surfaces that absorb or adsorb moisture, which also depends on the environmental temperature and humidity. It is therefore expected that the surface will play an important role in determining the magnitude of the tip–sample forces in viscoelastic materials, which are generally “soft” and for which the indentation is relatively large. For all these reasons, it is not possible to fully describe the behavior of the surface using elastic–dissipative models in which the internal forces are always normal to the surface, even if they exhibit the correct bulk viscoelastic behavior as in the case of the previously introduced Q3D model (based on the SLS).

## Results and Discussion

### Quasi-three-dimensional SLS model with surface effects

**Figure 6:**
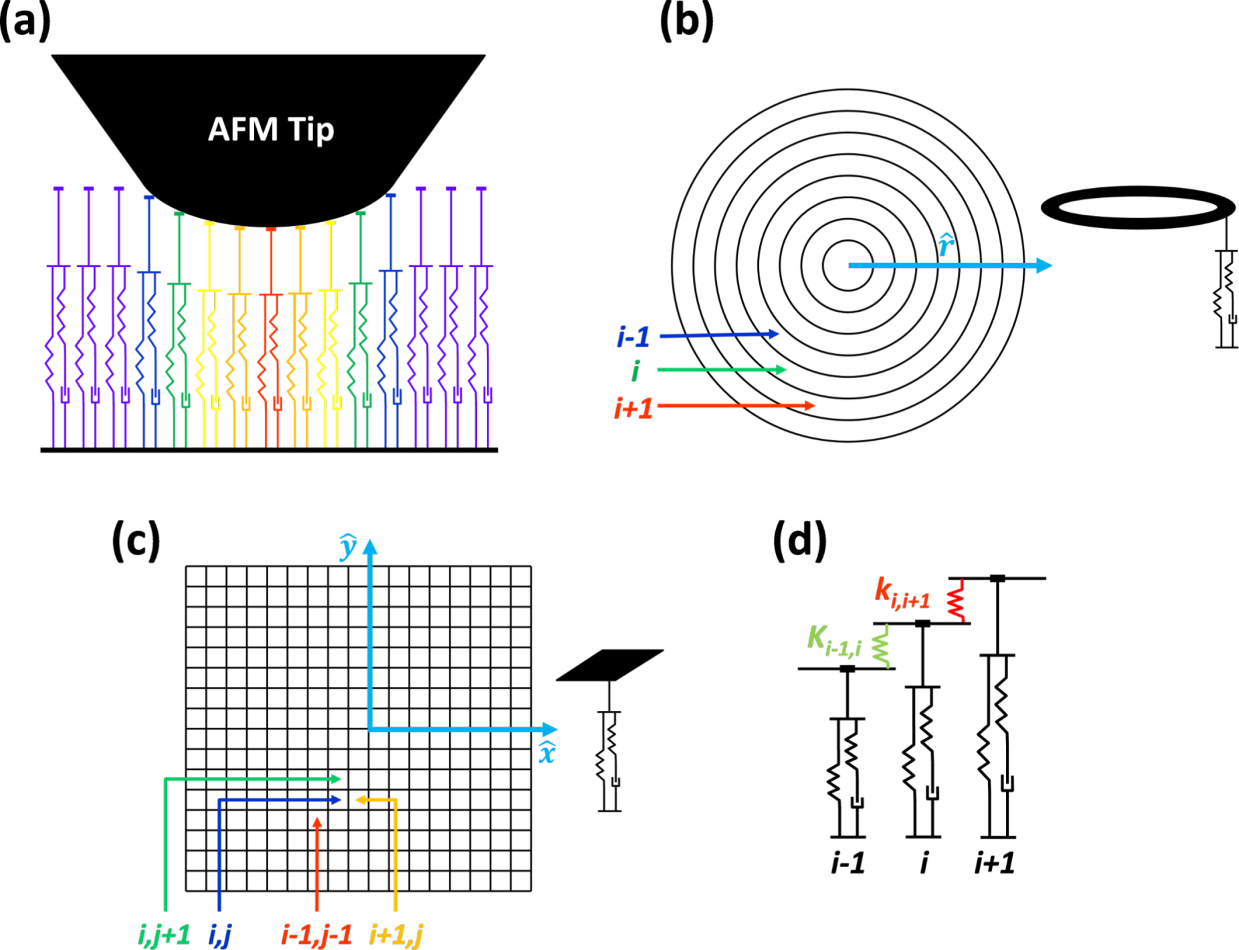
(a) Schematic representation of the Q3D sample model interacting with the AFM tip. (b) Polar coordinates partition of the surface illustrating an individual element (here the SLS parameters of each element differ because their areas differ). (c) Cartesian coordinates partition of the surface illustrating an individual element. (d) Q3D model enhanced with inter-element springs to represent surface effects. In the polar representation of (d) used in this paper, the force constants between adjacent surface elements depend on the contact perimeter between each two elements.

**Figure 7:**
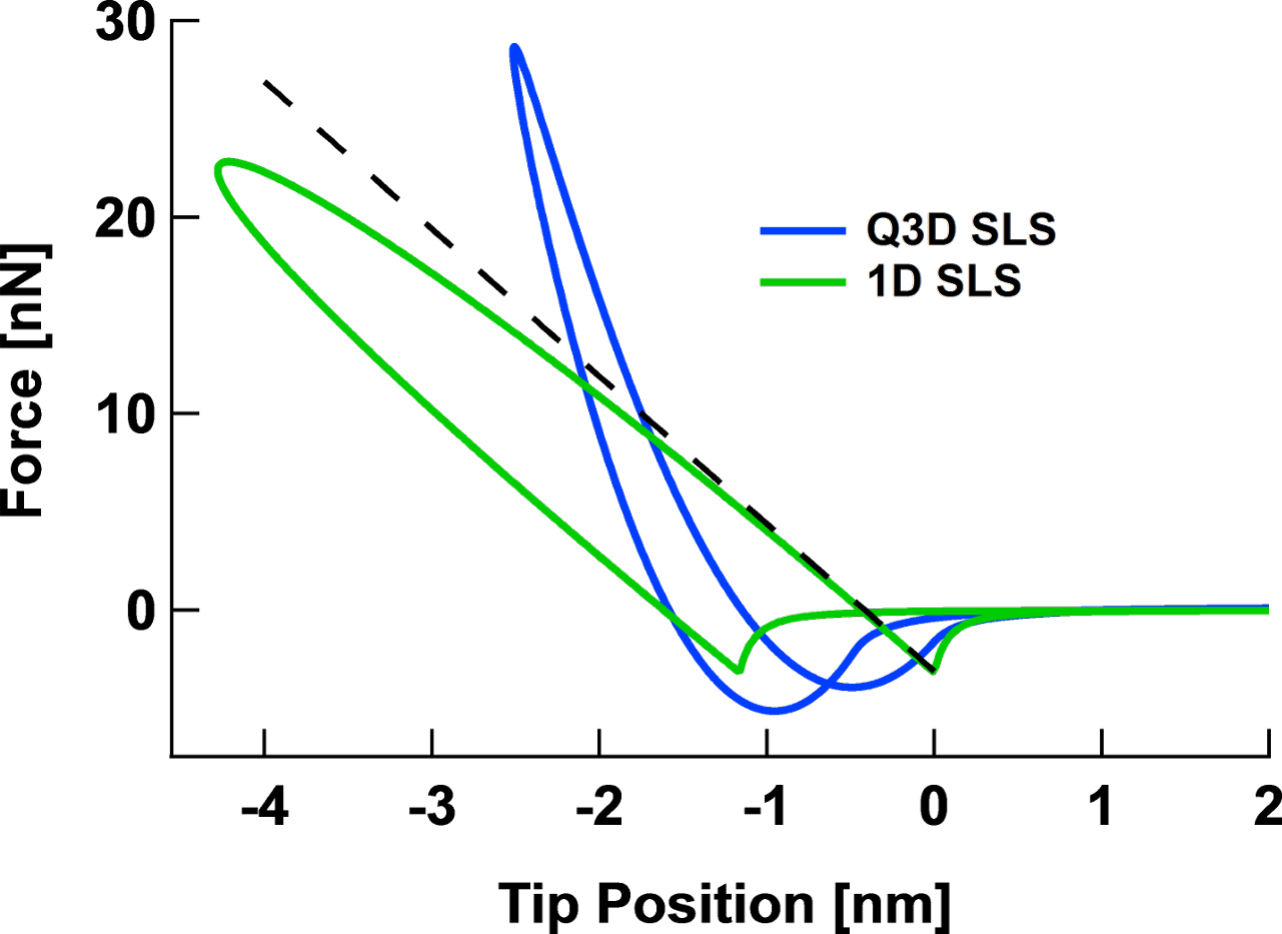
Comparison of typical force curves obtained for the Q3D model and the 1D SLS model in AFM simulations. The force curves for the latter do not exhibit the correct curvature behavior in the repulsive force region.

A previous publication [22] introduced a quasi-three-dimensional (Q3D) simulation software implementation of the SLS model. In this work, the surface is partitioned into very small area elements, each of which can undergo displacements in the direction normal to the surface upon interaction with the AFM tip. Figure 6 shows a conceptual representation of the model, with area elements partitioned either in the radial direction to simulate AFM imaging with axisymmetric tips (Figure 6b) or partitioned in the *x-* and *y-*directions for general tip shapes (Figure 6c). Within this model, as the tip penetrates deeper into the surface, it interacts with a larger number of viscoelastic elements. This leads to a repulsive tip–sample force curve that exhibits an upward curvature instead of the downward curvature corresponding to the previously used 1D SLS model [14,28] (see Figure 7). Additionally, since within the Q3D model the tip creates a cavity on the surface of the material, the total van der Waals force experienced by the tip depends on the geometry of that cavity, or more specifically, on the number of surface elements that are in close proximity to the tip surface [22]. This is why the maximum attractive force differs for the approach and retract of the Q3D force curve of Figure 7 (note that the model does not currently include other types of forces, besides the force due to the viscoelastic elements and the van der Waals forces).

Since the Q3D model is based on individual 1D SLS elements, it also exhibits the qualitatively correct behavior with respect to creep and stress relaxation. However, in its previous form [22], it lacks interaction between adjacent area elements, and thus, it does not produce a physically correct shape for the surface profiles that emerge upon indentation by the AFM tip. This is because the only area elements that undergo displacement are those directly under the tip, which the tip directly depresses. Thus the Q3D model (in its polar coordinates implementation) has been enhanced by introducing additional linear springs between the adjacent concentric area elements (Figure 6d), which can be loosely related to surface free energy or elasticity. Due to the fact that the area elements in the given model only relax in the vertical direction and not in the horizontal direction, the connection to surface effects is most easily understood in terms of elasticity. In this case, the restoring force can be modeled through an inter-element force constant for the surface, *k*_s_, such that the force between adjacent area elements, *i* and *i* + 1, displaced by a distance d*z* with respect to one another, is equal to

[21]
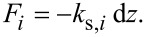


This equation includes the subindex *i* in *F_i_* and in k_s,i_ to indicate that the force constant between two area elements varies with the distance between those elements and the vertical axis of the tip (radial coordinate origin). Additionally, *k*_s_*_,i_* can be approximately related to the 2D elastic properties of the surface via a 2D stress–strain relation:

[22]
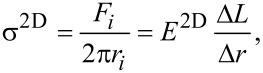


where *E*^2D^ is the 2D Young’s modulus of the surface, Δ*r* is the unstrained width of each area element (element size in the partition of the surface area), and 2π*r_i_* is the perimeter of element *i*, located at a distance *r_i_* from the vertical axis of the tip. Additionally, Δ*L* is the increase in the width of the area element, which is proportional to d*z* via a trigonometric factor of order unity. Therefore, the force constant is approximately equal to

[23]
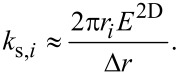


For simplicity, in the code provided, the user enters a cohesiveness parameter equal to

[24]
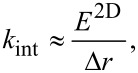


which can be easily related to *k*_s,_*_i_* via Equation 23 once Δ*r* is defined (note that Δ*r* can take different values in the code, depending on the desired fineness of the surface partition).

The incorporation of surface free energy effects is mathematically more complex because a simple order of magnitude approximation (as the one used above) is not possible. To see this, consider concentric area element *i*, which is displaced vertically with respect to element *i* + 1 by a distance d*z*. In this case, the increase in surface area d*A* is of the order d*A* ≈ 2π*r_i_*d*z* (again, within a trigonometric factor of order unity). This leads to an increase in surface free energy d*E* ≈ 2π*r_i_E*_s_d*z*, where *E*_s_ is the specific surface area (energy per unit area). This change in free energy, in turn, generates a force between the two area elements given for element *i* by

[25]
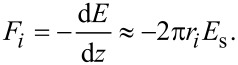


In the case of the polar coordinate system considered here, this increases linearly with the distance from the origin, but does not depend on the separation between the area elements, d*z* (the force acting on element *i* + 1 is of course equal to −*F_i_*). Such independence of the force with respect to the displacement (within the approximation considered) leads to discontinuous displacements of the surface area elements and thus gives unphysical results. A more refined approximation is possible by explicit consideration of the surface tilt angles of the various area elements, but that approach results in a much more complex and less efficient calculation. Since this more complex calculation would still not account for the physics of the problem quantitatively due to the limitations inherent to the model (fixed area element width and restriction to motion in the vertical direction), it was not incorporated into the current code.

The enhanced Q3D model is limited in various ways, each of which offers a research opportunity. For example, the presented viscoelastic treatment of the subsurface only considers tensile (compressive) strains, thus neglecting shear. This is common practice in AFM simulation, where 1D models are the norm (e.g., [2–3,10,12–14,16,28]), but neglects the fact that indentation of the tip into a soft material introduces surface curvature and subsurface 3D strain, which can become important as the tip wedges itself further into the material. A second important limitation is that the model offers only a continuum approximation of the sample, which ignores lateral variations in local morphology and topography (see Figure 4), as well as molecular and specimen dimensions. A third shortcoming of the approach presented is that it is based on linear viscoelasticity, which may not be applicable when large forces are rapidly applied to the surface, as in intermittent-contact multifrequency AFM methods [9,28]. In fact, the treatment of the in-plane surface forces is so far only linear elastic. A fourth, related, limitation is that the model assumes uniform material relaxation in the subsurface, which cannot be guaranteed even in one dimension. Adhesion forces other than attractive van der Waals forces are also not considered, although these can be important and may even lead to noticeable upward deformation of the surface. Despite this already comprehensive list of limitations of the Q3D model, it may still offer a qualitative means to begin to develop a richer description of the physics of the surface and subsurface of viscoelastic materials in the specific context of AFM imaging simulation, especially as experimental results become available.

### General features of the tip–sample interaction force curve

It is shown in [22] that the previously introduced Q3D model qualitatively reproduces the correct repulsive region curvature and other features of the tip–sample force curve, such as the offset between the position of minimum (most attractive) force during the approach and its position during the retract (see Figure 7). The upward curvature of the force curve results from the fact that the number of surface elements interacting with the tip increases as the latter penetrates deeper into the surface. For a spherical tip, for example, each SLS element interacting with the tip follows a time-dependent relaxation similar to that of a simple SLS element [14,22], with a time delay for the onset of deformation and with decreasing indentation for each successive element in the radial direction. The offset of the force minima is a consequence of viscoelastic surface relaxation during the tip–sample contact period, as extensively discussed in previous publications [14,22]. The overall features of the force curve do not change when surface effects are introduced via the spring elements illustrated in Figure 6d, but significant differences can be observed in the quantitative behavior of the force curve and surface profiles, as discussed in the next section. Note that in this paper the viscoelastic behaviors of the models are primarily discussed in terms of the corresponding tip–sample force curves (force vs distance). However, it can also be instructive to examine the curves of the tip–sample force plotted against time. An extensive discussion of the qualitative features of such curves for single- and multiple-impact interactions within the SLS model is provided in [14]. The qualitative features of the curves corresponding to the models discussed here are similar.

#### Surface effects in the tip–sample interaction force curve

**Figure 8:**
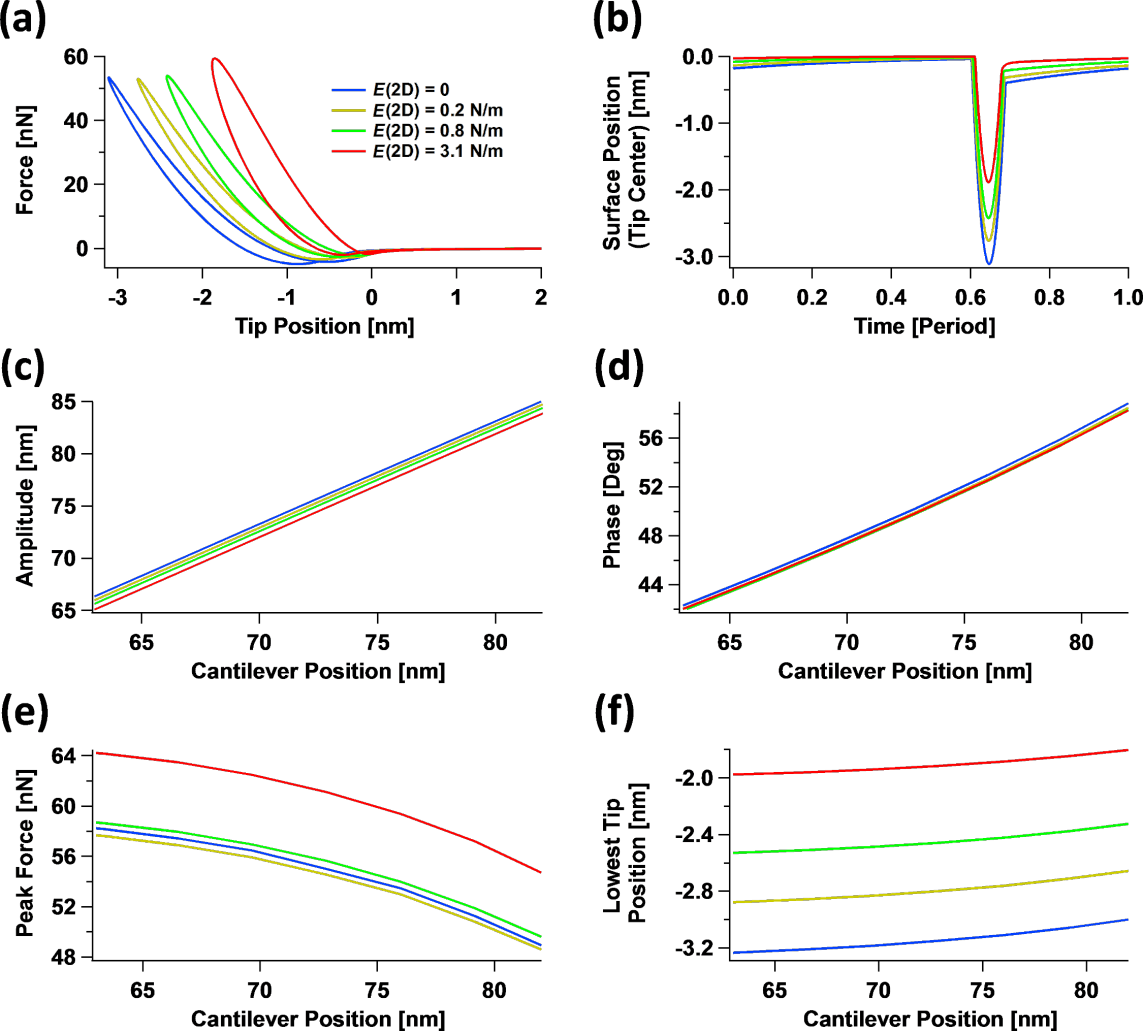
Effect of the 2D surface Young’s modulus on tapping-mode AFM: (a) tip–sample force curves; (b) indentation depth vs time (given in cantilever oscillation periods) for the surface element located directly below the center of the AFM tip; and (c–f) tip oscillation amplitude, fundamental phase shift, maximum tip–sample force observed during each cantilever oscillation, and maximum indentation of the surface element located directly below the tip center observed during each cantilever oscillation, respectively, for cantilever positions between 60% and 85% of the free oscillation amplitude (the simulation parameters are given in the text).

The simulations show that the inclusion of surface elasticity via force constants joining adjacent SLS surface elements (Figure 6d) does not necessarily change the overall shape of the force curve features. This, in turn, suggests that the fact that a particular experimental set of spectroscopy data may fit a given analytic function (e.g., a Hertzian curve where the force varies with respect to the indentation with an exponent of 1.5) does not guarantee that the interpretation of the physics is correct. Consider, for example, the results of Figure 8a, where force curves of similar overall appearance are obtained for different values of the 2D Young’s modulus of the top surface layer, with the same viscoelasticity parameters in the subsurface. Certainly the curves differ for different moduli, but their overall qualitative appearance is similar and they could be fit with similar analytic functions that do not consider the physics of the surface deformation. In fact, one may be inclined to attribute the variation in the steepness of the curves to variations in bulk elasticity. This would seem to be consistent with the reduced indentation observed (assuming it could be measured) and some small variation in the phase and amplitude of the cantilever oscillation. For the various values of the 2D Young’s modulus considered, Figure 8 also illustrates the time-dependent relaxation behavior of the surface element directly under the tip (Figure 8b), the oscillation amplitude and phase (Figure 8c and Figure 8d, respectively), the peak force within one fundamental oscillation (Figure 8e), and the maximum indentation within one fundamental oscillation (Figure 8f).

The cantilever parameters used in Figure 8 were as follows: resonance frequency 150 kHz, force constant 10 N/m, quality factor 150, free oscillation amplitude 100 nm, and tip radius of curvature 20 nm. The cantilever position above the sample was 76 nm for (a) and (b). The subsurface parameters were as follows: 
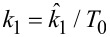
 = 0.075 N/m·nm^2^, 
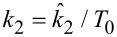
 = 0.075 N/m·nm^2^, and 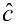 = 1 × 10^−7^ N·s/m·nm^2^. The 2D (in-plane) surface Young’s modulus *E*^2D^ is indicated in the legend on the graph in Figure 8a and the color coding of the traces is the same for all graphs. For reference, *E*^2D^ = 3.1 N/m corresponds to *k*_int_ = 2.4 × 10^10^ N/m^2^ and is approximately equivalent to 1/120 times the 2D Young’s modulus of graphene [32].

**Figure 9:**
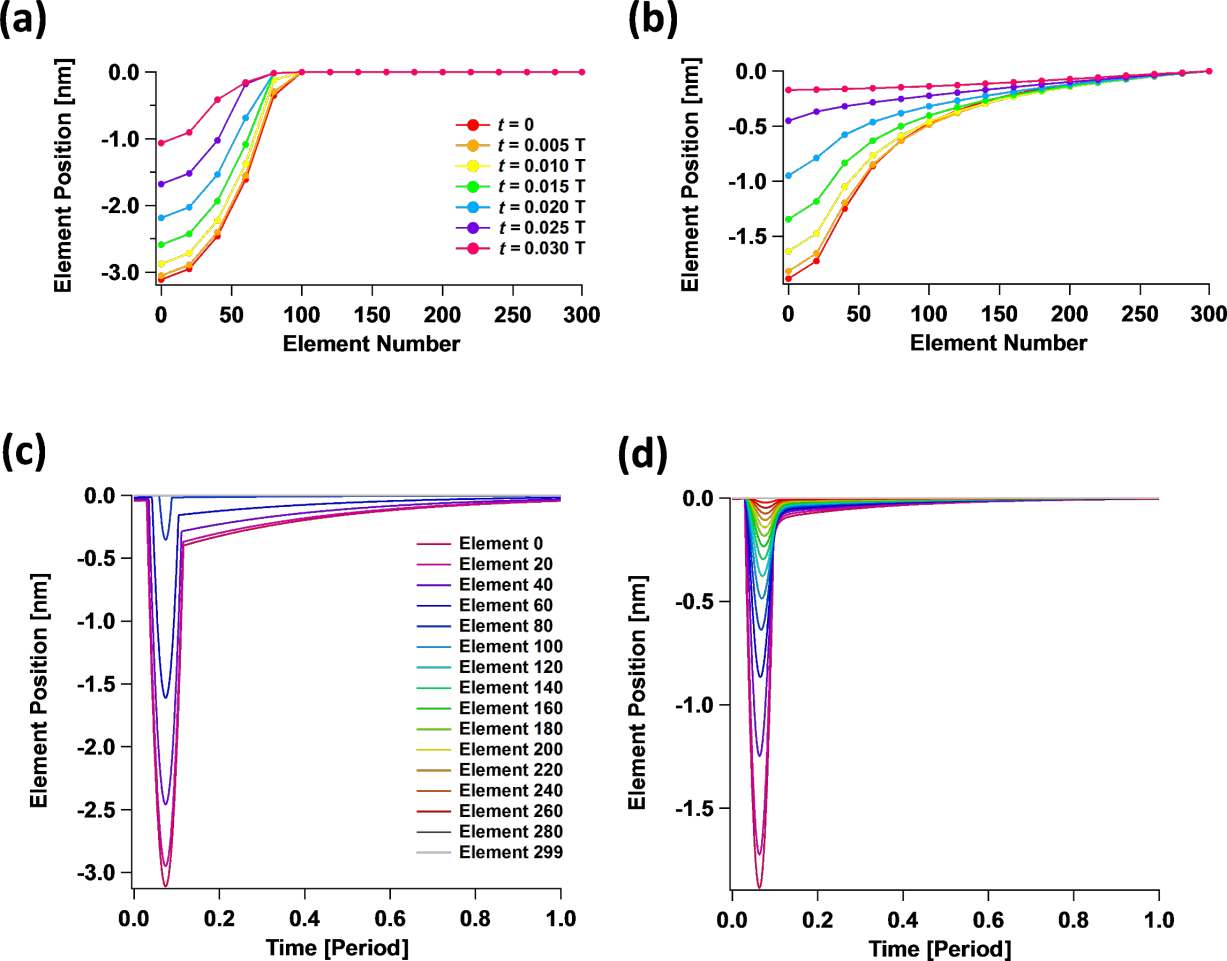
Surface indentation profiles corresponding to the simulations of Figure 8. (a) Indentation of the surface vs distance from the polar coordinate origin in the absence of in-plane surface interactions, for different times given in terms of the tip oscillation period (note how the indented region is limited to the area of direct tip–sample contact). (b) Indentation profiles analogous to those in (a) for a 2D surface Young’s modulus of 3.1 N/m. (c) Relaxation of the various surface elements plotted as indentation vs time for the same case as (a). (d) Relaxation of the various surface elements plotted as indentation vs time for the same case as (b). Notice that the relaxation of the various surface elements shown in (c) and (d) exhibits a tip–sample contact region (the deeper part of the plot, on the left) separated by a sharp change in slope from the region of viscoelastic creep, corresponding to relaxation after tip–sample contact has been interrupted. The legend of (b) is the same as that of (a), and the legend of (d) is the same as that of (c).

It is illustrative to examine in more detail the significant effect that a small variation of the 2D surface modulus can have on the results. For example, for the steepest curve in Figure 8b, the 2D modulus of the surface is only 3.1 N/m. If one considers an “effective” surface thickness of ≈1 nm, then one obtains a 3D Young’s modulus of approximately (3.1 N/m)/1 nm = 3.1 GPa. This value is comparable to the calculated rubbery and glassy moduli for this simulation, which were on the order of 3.75 GPa and 7.5 GPa, respectively (see previous section for an estimation of the rubbery modulus). The value of 3.1 N/m is particularly small if one considers that the in-plane surface displacements are expected to be small. However, the effect of including surface elasticity in the model is significant because it can drastically change the shape of the indentation profile (Figure 9), softening its curvature and extending the deformation well beyond the area of direct tip–sample contact. This, in turn, leads to the involvement of a larger number of subsurface SLS elements in comparison to the case when the surface elastic modulus is zero. Figure 9 compares the shape of the indentation profiles and their relaxation in time for the case of zero surface elastic modulus (Figure 9a) and a 2D surface elastic modulus of 3.1 N/m (Figure 9b). The latter corresponds to approximately 1/120 of the 2D Young’s modulus of graphene [32]. The profiles in Figure 9a and Figure 9b are “snapshots” of the indentation profile (position vs surface element number) observed for different times given as a function of the cantilever oscillation period, while the data in Figure 9c and Figure 9d provide the corresponding positions of the various surface elements along the radial direction, plotted individually as a function of time. In these simulations, the outermost surface element was kept fixed (element number 299, located at a radial distance from the polar coordinates origin equal to 1.9 × (tip radius) = 38 nm).

It is also worth emphasizing the counterintuitive observation that in Figure 8a the force curves corresponding to a larger 2D surface elastic modulus (which Figure 8f indicates lead to shallower indentations) exhibit the largest amount of dissipation (they have hysteresis loops of larger area [14]). This is a consequence of the fact that the deformation area is larger when in-plane surface effects are included. This leads to the recruitment of a larger number of SLS elements in the subsurface, thus leading to a larger total dissipated energy. While the explanation is simple, this observation has very significant implications for the interpretation of experiments, for which it is customary to assume that larger amounts of dissipation are expected for samples that are “more viscous” and/or “less elastic”. However, the above simulations suggest that the correlation between dissipation and bulk properties can be significantly influenced by in-plane surface forces.

A final subtle observation can be made based on Figure 8c, which indicates that in-plane surface effects lead to changes in the tip oscillation amplitude. This is not unexpected, but is a reminder that topographical measurements in AFM are subject to errors due to changes in material properties. In the case under consideration, the relaxed surface is located at the same position with respect to the cantilever for all simulations labeled with the same cantilever height. However, changes in the surface properties can lead to changes in the amplitude on the order of 1–2 nm for the parameters considered, which will give a comparable error in the topographical measurement.

#### Interaction between 2D surface elastic modulus and tip geometry

**Figure 10:**
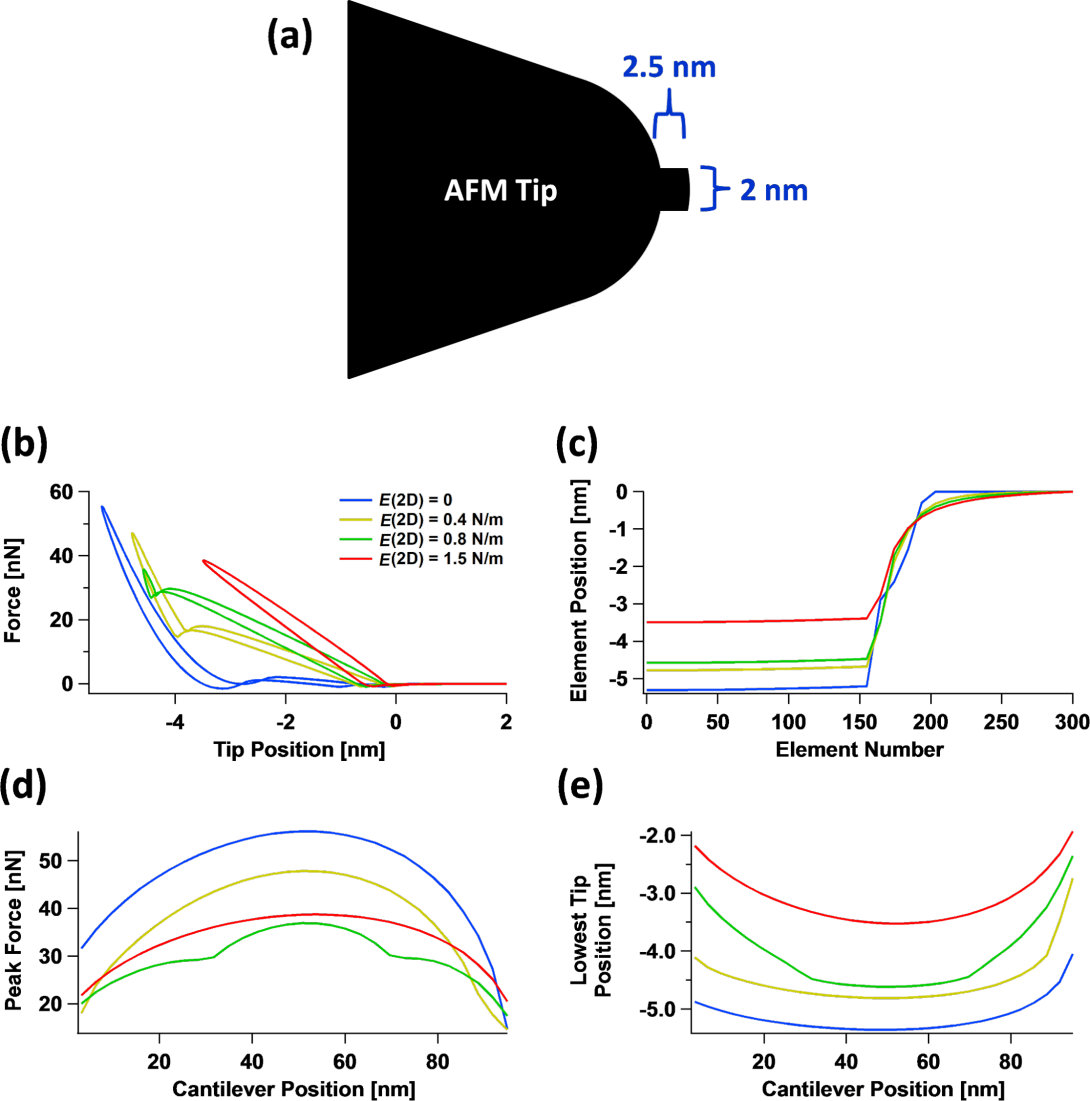
Simulation of the interaction of an AFM tip having an irregular protrusion with surfaces of varying 2D Young’s modulus: (a) AFM tip geometry; (b) force curves; (c) surface indentation profiles; (d) peak force observed during one full oscillation of the cantilever versus cantilever position; (e) maximum indentation observed during one full oscillation of the cantilever versus cantilever position. *E*^2D^ is indicated in the legend of (b) and also applies to graphs (c–e).

One of the most relevant consequences of having different properties at the surface in comparison to the bulk material is that the interaction of the surface with specific tip geometries becomes more complex. This is illustrated in Figure 10 for an irregular tip with a protrusion interacting in tapping mode with four surfaces having different values of the 2D surface modulus of elasticity. The force curves (Figure 10b) vary greatly for all four cases not only in simple ways such as steepness differences, but also in overall shape (the corresponding indentation profiles are given in Figure 10c). This happens because there is a gradual evolution between a regime in which the surface is able to interact with both the tip protrusion and the rest of the tip (this happens when in-plane surface elasticity is weak or nonexistent [22]) to a regime in which only the protrusion interacts with the surface (this happens for large in-plane surface stiffness). Notice that there can be a transition between the two regimes as the cantilever position changes, as can be observed by inspection of the green trace in Figure 10d (peak force) and Figure 10e (maximum indentation). It is interesting to observe also that the order of the traces in Figure 10d is not the same as that of Figure 10e (the order of the red and green traces is inverted, in agreement with the force versus distance curves of Figure 10b). Additionally, Figure 10b shows that as the in-plane surface stiffness increases, the force curves resemble those obtained for the 1D SLS model (see Figure 7), since the effect of increasing contact surface area between the tip and the sample with increasing indentation is greatly diminished.

Notice that the peak forces of the green trace in Figure 10d are lower than those of the red trace, despite the fact that the indentation is greater for the green trace, as shown in Figure 10e. This occurs due to the attractive van der Waals interactions between the surface and bulk of the AFM tip, which partially offset the repulsive forces. The simulation parameters are the same as for Figure 8, except for the tip geometry, the 2D Young’s modulus values indicated in the legend of Figure 10b, and the cantilever positions indicated on the horizontal axis of Figure 10d,e.

**Figure 11:**
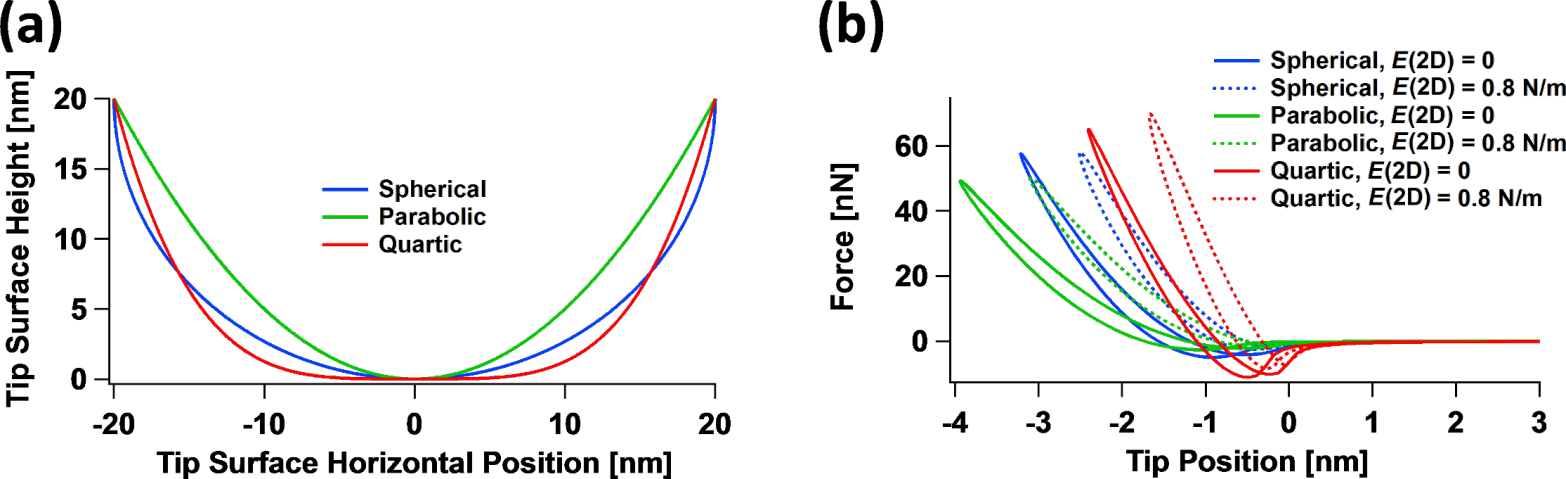
Interaction of tip curvature effects with in-plane surface elasticity effects: (a) three different tip surface profiles (spherical, parabolic and quartic); (b) tip–sample interaction force curves obtained for the profiles in (a) for two different values of the surface 2D Young’s modulus. Notice that the magnitude of the maximum attractive force upon approaching the surface differs among the profiles considered because each results in a different proximity between the surface area elements and the tip surface. The simulation parameters, other than the tip profiles shown in (a), the 2D Young’s modulus values indicated in the legend of (b) and the cantilever position (located 66 nm above the sample), are the same as for Figure 8.

The results of Figure 10 are instructive, but it could be argued that the tip irregularity considered is not common, and although not all tips are perfect, their profiles are often close enough to spherical. However, the simulations show that even in the case of relatively small differences in the tip profile, one can expect significant differences in the tip–sample interaction dynamics. To illustrate this, Figure 11 considers three different tip profiles (Figure 11a), namely spherical, parabolic and quartic. One would expect the variations in the interactions to be relatively small, especially when comparing the spherical and quartic profiles (the parabolic profile is a bit sharper than the other two), but Figure 11b shows that the force curves vary significantly both in steepness and in shape as the in-plane surface elasticity changes. It is interesting to note that not only the steepness and the area of the dissipation hysteresis loop changes, but also the maximum attractive force (“well depth” of the curves) can vary. This is because different tip profiles lead to different proximity between the surface elements and the surface of the tip, thus altering the overall attractive van der Waals interactions. These results suggest that even relatively mild deviations from ideality in the tip shape are important if one wishes to carry out quantitative material property measurements.

The interaction of surface mechanical behaviors with tip geometry effects suggests that the exact tip geometry should not be ignored in a quantitative measurement. Thus interpretations of AFM observables based on 1D models for which the effect of tip geometry can be eliminated from the equations may in some cases lead to an incomplete and, depending on the sample properties, even an incorrect interpretation of the physics.

**Figure 12:**
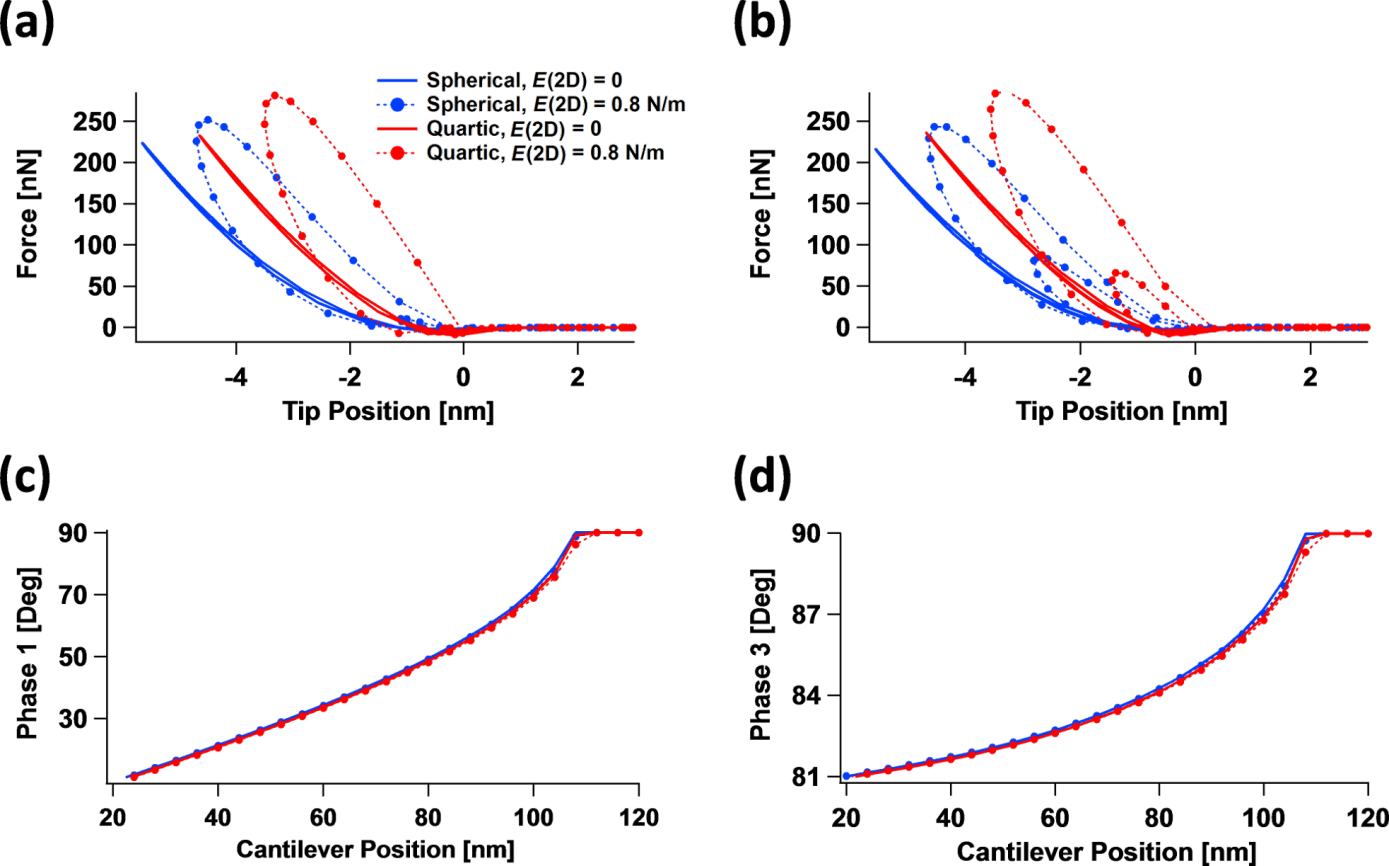
Interaction of tip curvature effects with in-plane surface elasticity effects for bimodal AFM using the first and third eigenmodes for the tip profiles shown in Figure 11a and the 2D surface Young’s modulus values indicated in the legend of (a) (same for all graphs): (a) tip–sample interaction force curves for a cantilever position of 80 nm above the surface; (b) tip–sample interaction force curves for a cantilever position of 64 nm above the surface; (c) phase of the first eigenmode vs cantilever position; (d) phase of the third eigenmode vs cantilever position. The sample and first cantilever eigenmode parameters are the same as for Figure 8, except for the cantilever positions given above and in the axes of (c) and (d). The third eigenfrequency was set to 2.64 MHz with a force constant of 3.1 kN/m and the third eigenmode’s free amplitude was set to 10 nm.

One final case to consider is the interaction between different tip geometries and the 2D surface Young’s modulus within multifrequency AFM imaging [9]. As has been previously discussed [14,28,33], the interaction of the tip with the sample becomes much more complex in this case because multiple tip–sample impacts may occur within a single cycle of the dominant cantilever oscillation (generally the fundamental eigenmode oscillation). A striking example of the complexities and limitations involved is offered by Figure 12. Shown here are multifrequency AFM tip–sample force curves for the same free oscillation amplitude of the first (100 nm) and third eigenmodes (10 nm) for two different positions of the cantilever (80 nm for Figure 12a and 64 nm for Figure 12b), for the spherical and quartic tip profiles of Figure 11. The curves exhibit the expected shape and features, with double-impact loops being clearly discernible in Figure 12b, as well as the expected differences between the various cases considered. The clear differences observed in the nature of the tip–sample interactions are a consequence of intentional variations chosen for the 2D surface Young’s modulus and for the tip profiles. However, it may be unexpected that the phase curves for the first eigenmode (Figure 12c) and for the third eigenmode (Figure 12d) are so similar to one another. In fact, the curves are hard to distinguish and may be indistinguishable in a real experiment where noise and other limitations may obscure the differences. This example thus highlights one of the most serious limitations in AFM spectroscopy characterization of nontrivial surfaces: the number of observables, even in multifrequency AFM, can be much smaller than the multiple degrees of freedom that describe the sample’s material behavior. The latter may include degrees of freedom related to surface elastic or viscoelastic effects as well as subsurface viscoelastic effects, which may be linear or nonlinear. Each of these effects may require more than one parameter for a proper description, even for each individual Cartesian coordinate (in fact, the 1D SLS model, which is only a linear model, already requires three parameters!). Additionally, the various degrees of freedom of the sample may exhibit different interactions with the tip geometry and imaging parameters. Recall that the number of imaging parameters that the user can vary generally increases with the sophistication of the method; for example, bimodal AFM requires more imaging parameters than single-mode amplitude-modulation AFM [34].

With regards to the complexity in the tip–sample interactions (described in the previous paragraph for multifrequency AFM), it is worth noting that although tip–sample impacts are in principle much simpler for single-frequency AFM, during a scanning experiment, there is an interplay between scanning speed, cantilever oscillation frequency and the recovery time of the surface, such that tip–sample impacts may occur at a location that has not fully relaxed from a previous impact, thus leading to very complex interactions. In general, an experimental viscoelastic study involving intermittent-contact dynamic modes of AFM should consider the physical horizontal distance on the surface between the locations of two successive impacts in order to assess whether each impact can be assumed to occur on a fully relaxed region of the sample.

#### Software tool description

The software tool used for the simulations reported here was written in standard C programming language and is provided as supplementary information. The program performs a spectroscopy curve similar to the program provided with [22], except for the introduction of the 2D surface Young’s modulus for the description of in-plane surface forces, as described in the first section of the Results and Discussion above. The code is based on trimodal excitation of the cantilever, whose simulation has been described in previous publications [14,22–23], and is thus not repeated here. The code uses constant-frequency, constant-amplitude excitation of the active eigenmodes, although other schemes or imaging modalities, such as scanning, can be implemented.

## Conclusion

A simulation study has been carried out to gain insight into the mechanical behaviors exhibited by viscoelastic materials in the context of AFM imaging. The study is based on an enhanced version of a previously developed quasi-three-dimensional model that treats the surface as a collection of standard-linear-solid viscoelastic elements. It additionally considers the existence of in-plane surface forces via an approximate inclusion of a 2D Young’s modulus for the top surface layer. A discussion of linear viscoelasticity concepts is also provided. Simulations are presented for single- and multifrequency intermittent-contact AFM imaging, whereby the corresponding surface indentation profiles and tip–sample interaction force curves, as well as their implications in the context of experimental interpretation are discussed. Where appropriate, discussions relevant to contact-mode-based methods such as contact-resonance and force-modulation AFM are included. A variety of intuitive, but not previously addressed phenomena are examined, which highlight the need for further research in the development of more physically accurate sample models for simulation and interpretation of AFM experiments. Finally, a software tool written in C programming language is provided as supporting information. The software can easily be used by individual researchers or could also be incorporated into existing simulation platforms that offer public access, such as VEDA [35] and dForce [36].

## Supporting Information

File 1 Description of software files provided.
Overview of software files content and usage.

File 2 Trimodal AFM with quasi-3D standard linear solid model sample, with in-plane surface effects added.
Software source file written in C programming language.

File 3 Parameter input file.
User-defined input parameters.
