# Supplementary material for: Nanoscale effects in the characterization of viscoelastic materials with atomic force microscopy: coupling of a quasi-three-dimensional standard linear solid model with in-plane surface interactions
Source: Beilstein J Nanotechnol. 2016 Apr 15;7:554–71. doi: 10.3762/bjnano.7.49 (PMC4901551; doi:10.3762/bjnano.7.49)
Supplement: File 1 — Description of software files provided. Overview of software files content and usage. [file Beilstein_J_Nanotechnol-07-554-s001.pdf]

Supporting Information

for

Nanoscale effects in the characterization of viscoelastic materials with atomic force microscopy: coupling of a quasi-three-dimensional standard linear solid model with in-plane surface interactions

Santiago D. Solares*

Address: Department of Mechanical and Aerospace Engineering, George Washington University, Washington, DC 20052, United States

Email: Santiago D. Solares - ssolares@gwu.edu

* Corresponding author

Description of software files provided

The software tool provided consists of the following two files:

1. “**Trimodal_AFM_With_Enhanced_Q3D.c**”(source code written in standard C language, ready to be compiled) contained in S2.zip and
2. “**input.txt**” (input file) contained in S3.zip.

In order to execute the code, it is necessary to compile the first file to create the executable, and place the second file in the same directory as the executable. The user may edit the file ‘input.txt’ in order to provide the desired simulation parameters to the program.

Content of the input file

The input file allows the user to specify the following parameters (the units are given in parenthesis when applicable):

1. **OutputFile:** Root name for the output files.
2. **Frequency_1 (Hz):** Cantilever fundamental eigenfrequency.
3. **ForceConstant_1 (N/m):** Cantilever fundamental force constant.
4. **QualityFactor_1:** Fundamental eigenmode quality factor.
5. **QualityFactor_2:** Second eigenmode quality factor.
6. **QualityFactor_3:** Third eigenmode quality factor.
7. **CantileverRestPosition (nm)**: Starting rest position of the cantilever in the construction of the spectroscopy curve.
8. **TargetAmplitude_1 (nm):** *Free* amplitude of the fundamental eigenmode.
9. **TargetAmplitude_2 (nm):** *Free* amplitude of the second eigenmode.
10. **TargetAmplitude_3 (nm):** *Free* amplitude of the third eigenmode.
11. k_1 **(N/m/nm^2):** Residual spring constant of the SLS model (see Figure 1 of the main paper).
12. k_2 **(N/m/nm^2):** Second spring constant of the SLS model (see Figure 1 of the main paper).
13. c_{Diss} **(Ns/m/nm^2):** Dashpot constant of the SLS model (see Figure 1 of the main paper).
14. k_{int} **(N/m^2):** Surface 2D inter-element elasticity parameter (see section 4 of the main paper).

In addition, the user may want to modify the following variables in the code (the location is indicated in parentheses):

1. **TipRadius (‘CONSTANTS’ section at the beginning of the code):** Radius of curvature of the AFM tip in nm.
2. **PartitionSize (‘CONSTANTS’ section at the beginning of the code):** Variable defining the number of area elements used in the surface partition.
3. **NoPoints (‘CONSTANTS’ section at the beginning of the code):** Number of points (different cantilever positions) desired in the spectroscopy curve.
4. **VdwStrength (‘CONSTANTS’ section at the beginning of the code):** Variable defining the strength of the van der Waals interaction between the tip and surface, in nN/nm^2.

5. **timestep (main program):** Timestep for the integration of the cantilever dynamics and relaxation of the surface.
6. **startprint (main program):** Time at which printing of the output files begins, defined in terms of the fundamental quality factor and period.
7. **printstep (main program):** Time increment at which the data in the output file is printed, defined in terms of the fundamental period.
8. **length (main program):** Total simulation time for each run, at each value of the cantilever position in the construction of the spectroscopy curve, defined in terms of the fundamental quality factor and period.

Output files

The software performs a spectroscopy curve simulation in which the cantilever is set at successively lower heights and driven while it interacts with the surface, until it has reached steady state at each height (this condition is defined by the variable 'startprint'). The calculation of the phase and amplitude begins two averaging periods earlier than 'startprint' in order to ensure that the calculated values are correct. The averaging time for the phases and amplitudes is defined in the code through the variable NN, which is currently set to a value of 25 (the averaging time for the fundamental eigenmode phase and amplitude is equal to NN times the fundamental period).

The program creates two output files for each equilibration of the cantilever at each fixed height. The name of the first file consist of the user-defined file root name plus the word 'Forces' and the name of the second file consists of the user-defined root name plus the word 'Profiles.' Additionally, the corresponding cantilever height is appended to the name of both input file names.

The **'Forces'** output file contains the following information printed with a simulation time interval given by the variable 'printstep' as defined in the code:

1. **time (ns):** Simulation time.
2. **Zeq (nm):** Cantilever position above the surface (the current code does not have any control loops, so this position remains constant throughout the simulation, but its output maybe useful in cases where specific imaging modes are implemented).
3. **Zt_m1 (nm):** First eigenmode instantaneous position.
4. **Zt_m2 (nm):** Second eigenmode instantaneous position.
5. **Zt_m3 (nm):** Third eigenmode instantaneous position.
6. **TipPos (nm):** Instantaneous tip position above the surface.
7. **Xb_SpecifiedPosition (nm):** Instantaneous position of the surface skin for a user-defined surface element (currently the code prints out the position of the zeroth surface element, directly below the tip, but this can be easily modified in the source code).

8. **Xc_SpecifiedPosition (nm):** Instantaneous position of the dashpot (damper) for a user-defined surface element (currently the code prints out the position of the zeroth surface element damper, directly below the tip, but this can be easily modified in the source code).
9. **TotalForceSLS (nN):** Total force exerted by the surface on the AFM tip.
10. **Amp1* (nm):** Instantaneous oscillation amplitude of the first eigenmode.
11. **Amp2* (nm):** Instantaneous oscillation amplitude of the second eigenmode.
12. **Amp3* (nm):** Instantaneous oscillation amplitude of the third eigenmode.
13. **Ph1* (degrees):** Instantaneous phase shift of the first eigenmode.
14. **Ph2* (degrees):** Instantaneous phase shift of the second eigenmode.
15. **Ph3* (degrees):** Instantaneous phase shift of the third eigenmode.
16. **Force_0 (nN):** Force contribution to the total tip-sample force from the zeroth surface element (it can be illustrative to compare the force contributions of different surface elements).
17. **Force_3 (nN):** Force contribution to the total tip-sample force from the third surface element.
18. **Force_6 (nN):** Force contribution to the total tip-sample force from the sixth surface element.
19. **Force_9 (nN):** Force contribution to the total tip-sample force from the ninth surface element.

*The various eigenmode amplitudes and phases do not change within a single impact, so their output is only relevant when the print period contains a large number of tip-sample impacts, in which case it can be used to assess the stability of the tip oscillation. Additionally, the phases are only meaningful for the driven eigenmodes.

The **'Profiles'** output file contains the instantaneous position of the surface skin (variable Xb) for surface elements 0, 20, 40, 60, 80, 100, 120, 140, 160, 180, 200, 220, 240, 260, 280, 299, printed with a simulation time interval given by the variable 'printstep' as defined in the code.

Finally, the program produces a third output file upon completing the entire spectroscopy curve, whose name consists of the user-defined root name plus the word **'Spectroscopy'** and which contains the following information recorded for all values of the cantilever height:

1. **Zcf (nm):** Cantilever position above the surface.
2. **Force (nN):** Maximum tip-sample force recorded during a period of the fundamental frequency.
3. **Depth (nN):** Maximum indentation recorded during a period of the fundamental frequency.
4. **Amp1 (nm):** Oscillation amplitude of the first eigenmode.
5. **Amp2 (nm):** Oscillation amplitude of the second eigenmode.
6. **Amp3 (nm):** Oscillation amplitude of the third eigenmode.
7. **Ph1 (degrees):** Phase shift of the first eigenmode.
8. **Ph2 (degrees):** Phase shift of the second eigenmode.
9. **Ph3 (degrees):** Phase shift of the third eigenmode.
